# Spread Spectrum Based Energy Efficient Collaborative Communication in Wireless Sensor Networks

**DOI:** 10.1371/journal.pone.0159069

**Published:** 2016-07-22

**Authors:** Anwar Ghani, Husnain Naqvi, Muhammad Sher, Muazzam Ali Khan, Imran Khan, Azeem Irshad

**Affiliations:** 1 Department of Computer Science & Software Engineering, International Islamic University, Islamabad, Pakistan; 2 NUST College of EME, National University of Science & Technology, Islamabad, Pakistan; Southwest University, CHINA

## Abstract

Wireless sensor networks consist of resource limited devices. Most crucial of these resources is battery life, as in most applications like battle field or volcanic area monitoring, it is often impossible to replace or recharge the power source. This article presents an energy efficient collaborative communication system based on spread spectrum to achieve energy efficiency as well as immunity against jamming, natural interference, noise suppression and universal frequency reuse. Performance of the proposed system is evaluated using the received signal power, bit error rate *(BER)* and energy consumption. The results show a direct proportionality between the power gain and the number of collaborative nodes as well as *BER* and signal-to-noise ratio (*E*_*b*_/*N*_0_). The analytical and simulation results of the proposed system are compared with *SISO* system. The comparison reveals that *SISO* perform better than collaborative communication in case of small distances whereas collaborative communication performs better than *SISO* in case of long distances. On the basis of these results it is safe to conclude that collaborative communication in wireless sensor networks using wideband systems improves the life time of nodes in the networks thereby prolonging the network’s life time.

## Introduction

The resource limited nature of sensor networks make the power source of a sensor node very crucial resource. The power source in these networks should be used intelligently as in many applications it is almost impossible to replace or recharge it [1].

Many approaches, like cooperative communication [2–4], Multihop routing [5], collaborative communication [6–8] and Beamforming [9–12], for sensible use of power source in sensor networks have been proposed. Each of these approaches have their own prose and cons for example in case of multihop system [5], communication over long distances may affect the power source of all the hops from source to destination specially in case of retransmission. Similarly in case of distributed beamforming schemes, source and cooperative relay nodes make sure that their signal be transmitted with such phases so that they can be constructively added at the destination. The issue with Distributed beamforming is it needs modification to the existing front end RF further increasing the complexity and cost of the system [4]. Many other techniques could be found in literature addressing the issues of energy efficiency from different perspectives like; energy efficient clustering and topology control [13, 14], node energy based polynomial distribution for improved routing [15], energy driven architecture (EDA) based on energy dissipation to minimize energy consumption in Wireless Sensor Networks [16] and many others.

This paper presents collaborative communication model for wireless sensor networks using Rayleigh fading and wideband channels to achieve energy efficient communication. In collaboration a set of transmitter nodes transmits the same data at the same time towards base station [17] to exploit spatial diversity in order to achieve different benefits. It is presumed that the data to be transmitted toward the base station is shared among collaborative nodes before the actual collaborative transmission starts may be using one the schemes in [6, 18]. Unlike cooperative communication where the transmitted information is relayed by intermediate cooperative nodes [3], in collaboration each node communicates directly with the base station without any intermediate relay node(s). From the study of the literature it is clear that if time, frequency and phase synchronization is achieved sensor networks, substantial gain in received power can be produced at the receiver [2, 6, 9–12, 19, 20]. However in collaborative communication high power gain can be achieved even with imperfect phase, frequency and time synchronization [7].

A simple motivation here is to combine the benefits of collaborative communication with the benefits of the wideband channels. The benefits of collaboration are explored in the literature as well as in our previous work [8] but in quite different channel scenario. A channel is wideband if the ratio of its bandwidth(W) and the information rate (R) is much greater than unity i.e, *B*_*e*_ = *W*/*R* ≫ 1, where *B*_*e*_ is known as the expansion factor [21]. Wideband channels offers benefits like immunity against jamming, interference and noise as well as using single frequency for all nodes (universal frequency reuse). These features are helpful in different applications of sensor networks, such as secure and uninterrupted information transfer from hostile environments like battle field or volcanic area. To the best of our knowledge, this article is the first to explore the combination of these two technologies and analyze the benefits they have offer together.

As a problem statement; for a given “N” number of collaborative transmitter nodes which transmit the same information to a common receiver (BS), this paper strive to design an energy efficient algorithm using collaborative communication in combination with wideband channels including the effect of *AWGN* noise and *Rayleigh* fading. It is aimed to achieve the following advantages: 1) Produce substantial power gain at the receiver. 2) Considerably mitigate the fading effect. 3) Study the trade-off between energy consumption and transmission distances.

Contribution(s) of this article are, a) derivation of mathematical model for collaborative communication (power gain, *BER* and energy consumption) with imperfect phase synchronization based on spread spectrum with noise and fading. b) Theoretical models shown that gain in received power grows with the number of collaborative transmitters *N* whereas the *BER* shows converse behavior with with signal-to-noise ratio (*SNR*). c) Derivation of theoretical model to compute the energy expense during communication among transmitter nodes themselves for synchronization as well as transmitters nodes and base station. d) Design an energy efficiency model based on the parameters of off-the-shelf products like, *CC2420* and *AT86RF212* [22, 23] as shown in Table 1. Transmission distance, number of transmitters nodes and phase error are taken into consideration while calculating the total consumed energy of the proposed system. “Break-even” distances are used in trade-off analysis between the power consumed in circuit operations and transmission power. “Break-even” distance is a point where power depletion of collaboration becomes equal to that of a *SISO* system. e) A technique to reduce the effect of fading using collaborative communication by achieving significant gain in the received power and reduce the required power to be transmitted by a factor N (for N is the number of transmitter nodes). f) The use of spread spectrum approach to mitigate the effect of noise, reduce natural as well as inter symbol interference and transmit information at even low power and single frequency so that it is immune to interference, jamming and eavesdropping.

**Table 1:** Details of off-the-shelf products with data and parameters used in simulation.

Symbol	Description	AT86RF212	CC2420
-	Modulation	BPSK	BPSK
*f*_0_	Operating frequency	915MHz	2.45GHz
*R*_*s*_	Transmission data rate (BPSK)	40Kbps	250Kbps
U	Operating voltage (typical)	3V	3V
*I*_*rx*_	Currency for receiving states	9mA	17.4mA
*P*_*rx*_	Receiving power, *P*_*rx*_ = *UI*_*rx*_	27mW	52.2mW
*I*_*idle*_	Currency for idle states	0.4mA	0.4mA
*P*_*cir*_	Electronic circuitry power, *P*_*cir*_ = *UI*_*idle*_	1.2mW	1.2mW
Ps	Receiver sensitivity	-110dBm	95dBm

This model avoid interference and noise thereby decreasing the number of bits in error. This leads to less number of retransmission as well as allow nodes to use low power for transmission of data thereby preserving their power source. Hence it prolongs the network’s lifetime by prolonging the life of a single node. It also improves the coverage range as the number of collaborative nodes increases. Therefor, collaborative communication is suitable for networks with limited resources like sensor networks. The theoretical expressions derived for received power and bit error rate are confirmed by simulation.

A comparison of the simulated and theoretical results show that collaborative communication in wideband channels is not only successful in mitigating the effect of fading but it significantly suppresses the noise power. As a result significant improvements have been observed in received signal power and *BER*. It is further revealed that an increase in the number of collaborative transmitters, increases the gain in received power whereas an increase in *SNR* lowers the *BER*. Results of energy efficiency model divulge a saving of 99% in energy consumption, due to the use of collaborative communication in a Rayleigh faded wideband channel, even if the signal is received out-of-phase.

In remainder of the paper Related work section presents a brief survey of related literature, system model section presents a list of assumption for the proposed system and a description and derivation of theoretical (mathematical) model for received power and bit error rate in a Rayleigh faded AWGN channel. Energy consumption has been discussed from the perspective of SISO and collaborative communication. Results and discussion section presents an analysis of the proposed system whereas conclusion section conclude the article.

## Related Work

It is evident from literature survey that wireless networks, if synchronized in phase, frequency and time, yield huge gain in the received power [24–27]. However it is also shown in different schemes [6, 7] that synchronization is not mandatory for achieving high gain in received power. Collaborative communication enables a system to achieve significant gain in received power even if the incoming signals are not synchronized in phase, frequency and time. Collaborative communication is basically a cooperation of different neighboring nodes to help forward a node’s data to a common receiver, called as base station. Collaborative communication for energy efficiency has been thoroughly investigated in [6, 7, 9], in Rayleigh fading channels with imperfect phase and frequency, assuming each node has only one scatter component which may not be the case in reality. It is also crucial that collaborative nodes are synchronization with each other. Many schemes have been proposed in the literature for synchronizing collaborative nodes prior to transmission of data towards the base station [28, 29].

The effect of time and frequency synchronization on collaborative communication, has been investigated in [30–33], to show what range(bounds) of frequency could be used. Two venues, efficient communication system and task scheduling through smart algorithms, for reducing energy consumption in wireless sensor networks has been explored in [34]. In both venues reduction in energy utilization has been claimed. Synchronization among collaborative nodes is more crucial than their distribution diversity. But it must be noted that synchronization among collaborative nodes can degrade system’s performance, in some groups of diversity distribution [11, 17, 20, 35]. In [6, 12], a *Quantized feedback* technique has been presented for wireless relay networks, not only to achieve synchronization but also a significant gain in the received power. An energy consumption model for *MIMO* has been proposed in [36, 37], where *SISO* and *MIMO* systems are compared. It has been concluded that *SISO* performs well when the distance between communicating node and base is small, whereas in case the distance between communicating node and base station is large, it is difficult for *SISO* to keep up with *MIMO* systems. A comparison of energy efficiency of virtual *MISO* and decode-and-forward is presented in [38], concluding decode-and-forward approach shows strong energy efficient behavior compared to virtual *MISO*.

**Fig 1:**
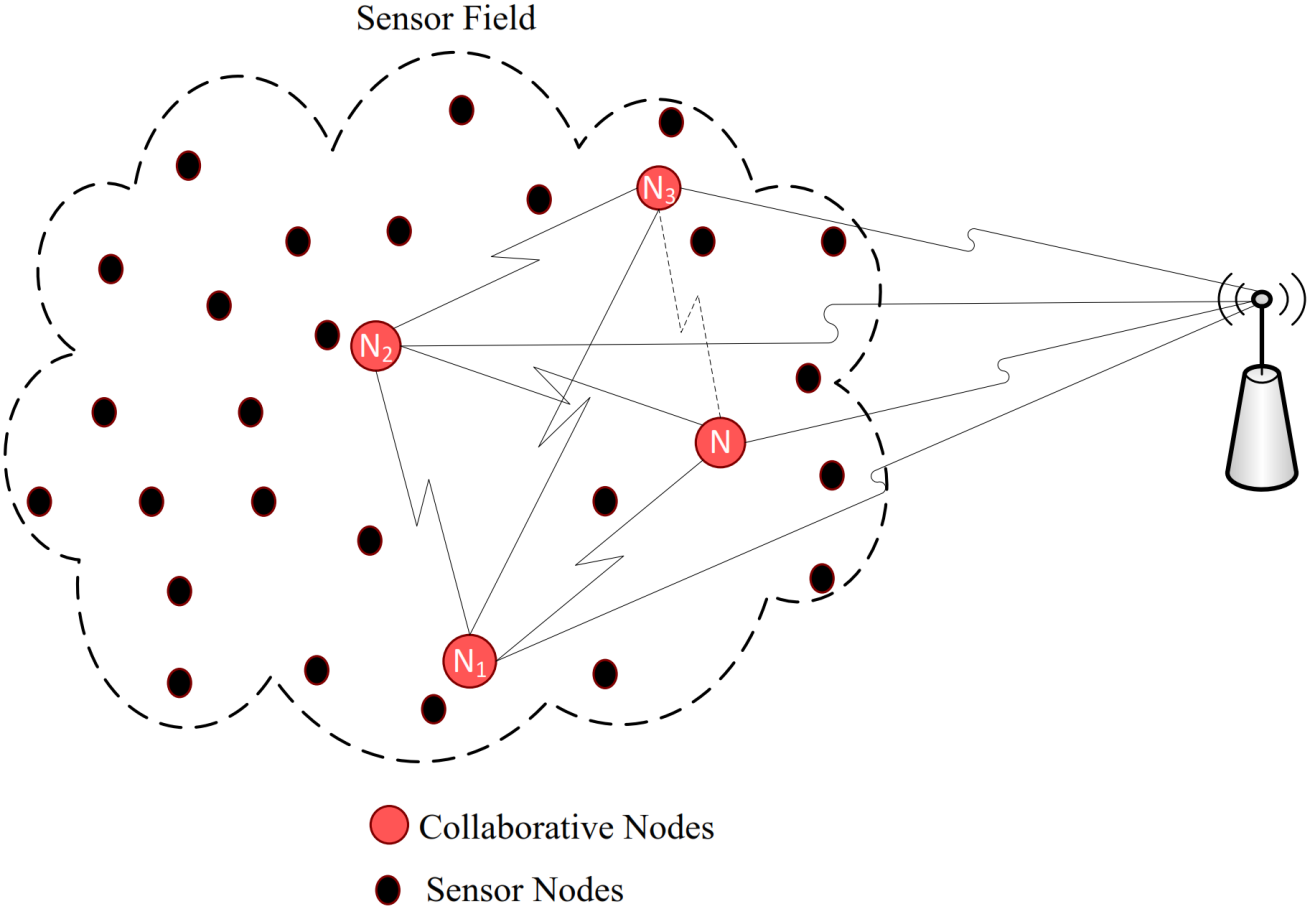
Geometry of sensor nodes.

The feasibility of applying cooperative *MIMO* system to *WSN* has been investigated in [39]. The authors argued that the cooperative *MIMO* systems are promising in terms low error performance, low power performance, tolerance to jitter and it outperforms other cooperative schemes in cases of imperfect synchronization. But it does not find the best or optimal scheme because they related diversity gain only with link reliability and do not consider the circuit power. A spread spectrum based communication system for range extension in wireless sensor network, to study the effect of collaboration has been investigated in [35]. The authors argued that using the natural diversity in collaboration; power from multiple sensor nodes can be combined to achieve robustness against the channel impairments and produce significant extension in the coverage range. Our approach however is to improve the energy consumption through collaboration in combination with spread spectrum by considering one receiver and multiple transmitter nodes from a sensor field as shown in Fig 1.

## System Model

### Assumptions

The assumption under which the proposed model is developed are listed as follow:

1. From a sensor field some randomly distributed (collaborative nodes) transmit the preshared (using one of the schemes [6, 18]) identical information concurrently to a common receiver(Base Station).
2. Single path communication is assumed here therefore, one signal component from each transmitter node using identical modulation is received subject to synchronization error making the system behave like virtual multipath fading system.
3. The noise in the channel is considered to be *AWGN*.

Unlike fixed array antenna systems, in distributed deployment it is often very close to impossible to determine the exact location of a node. Secondly there is no central network controller to synchronize the received signal therefore, the theory of fixed array antenna is not applicable [19, 40] directly. As a result there may be an estimation error in determining the position of a node in distributed deployment, known as the displacement error. Due to this displacement error the reception of the signals at the base station may be out-of-phase. So collaborative communication needs to synchronize the signal in time, frequency and phase to achieve power gain. In addition the use of spread spectrum provides the opportunity to exploit the transmitter diversity for achieving improved *SNR* values at the receiver [39].

As positions of the transmitters are not fixed in the proposed scenario and the estimation technique used to determine positions of the transmitters, gives most probable location rather than the exact location of a transmitter, therefor it is very difficult to fully synchronize the system. However, a general collaborative architecture where each node uses same carrier frequency based on spread spectrum approach, has been proposed in this article as shown in Fig 1. The collaborative nodes in the figure, are presumed to be synchronized with each other, before they begin information exchange with the base station.

### Theoretical Model

**Fig 2:**
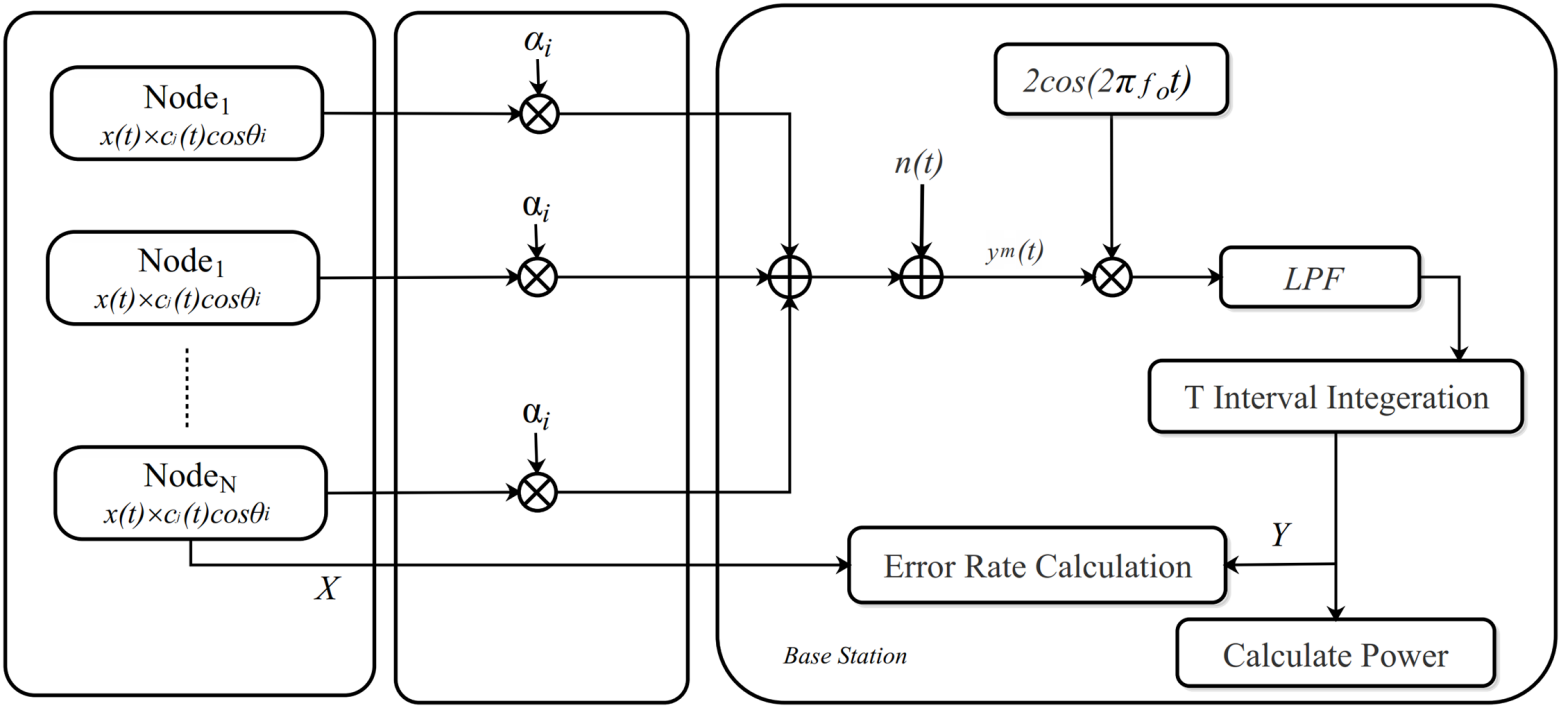
System model.

If in a random deployment *N* sensor nodes are considered in such a way that there is no “line-of-sight” between these *N* transmitters and the base station, for example in an urban or volcanic area. Therefore Rayleigh fading channel with *AWGN* is considered for the proposed system. A detail representation of the proposed system with *N* transmitter nodes is shown in Fig 2.

Each collaborative node transmits the same signal *x*(*t*) correlated with the chipping code *c*_*i*_(*t*) of length *M*. If “*d*_0_” is considered as the initial displacement of a node from the base station and *f*_0_ the carrier frequency, then the phase is given by “*θ*_0_ = 2*πf*_0_ *d*_0_/*c*”, where c represents the speed of light. Let the distance due to displacement error is *d*_*i*_, which when translated into phase error, produces a phase of “*θ*_*i*_ = 2*πf*_0_ *d*_*i*_/*c*”. If the carrier signal is “cos(2*πf*_0_ *t*)”, and not only the negligible signal delay in comparison to bit length lessens the chances of inter symbol interference but the use of spread spectrum approach fully mitigate this problem. The spread spectrum approach also exploits the transmit diversity to produce significant power gain without bandwidth expansion but at lower spatial rate.

The transmitted data *x*(*t*) from each node, is modulated using cos(2*πf*_*c*_ *t*) and correlated with the chip code *c*(*t*), to produce the transmitted data signal *s*(*t*) as follows.

$$\begin{array}{c} s\left(t\right)=\frac{1}{M}x\left(t\right)c\left(t\right)\cos\left(2\pi f_{c}t\right) \end{array}$$ (1)

where *M* is the length of chip code and *f*_*c*_ is the carrier frequency.

Signals from all *N* nodes are received at the base station with phase error as follow

$$\begin{array}{c} y\left(t\right)=\frac{1}{M}\sum_{i=1}^{N}\sum_{j=1}^{M}\alpha_{i}x\left(t\right)c_{j}\left(t\right)\cos\left(2\pi f_{c}t+\theta_{i}\right)+n\left(t\right) \end{array}$$ (2)

here *α*_*i*_ is the channel response/attenuation factor for *i*^*th*^ channel and *n*(*t*) is AWGN.

Signal in Eq (2) is demodulated, and decorrelated with chip sequence, yields the following result

$$\begin{array}{c} Y=\frac{1}{M}\sum_{i=1}^{N}\sum_{j=1}^{M}\alpha_{i}Xc_{j}\times c_{j}\cos\left(\theta_{i}\right)+n\times c_{j} \end{array}$$ (3)

here $X=\pm \sqrt{E}$ is the amplitude of the received signal whereas *n* represents noise amplitude.

Also we know from spread spectrum properties that $1/M\sum_{j=1}^{M}c_{j}\times c_{j}$ is equal to 1. Also let *P*_*Y*_ represents the signal power at the receiver (base station) therefore, the above equation can be rewritten as

$$\begin{array}{c} P_{Y}={\left[\sum_{i=1}^{N}\alpha_{i}X\cos\left(\theta_{i}\right)+\frac{1}{M}\sum_{j=1}^{M}n\times c_{j}\right]}^{2} \end{array}$$ (4)

Since *α*_*i*_, *θ*_*i*_, *n* and *c*_*j*_ are identically independent random (i.i.d), therefore the expected value of the above equation should be calculated in order to derive the power gain

$$\begin{array}{c} E\left[P_{Y}\right]=E{\left[\left[\sum_{i=1}^{N}\alpha_{i}X\cos\left(\theta_{i}\right)\right]\right]}^{2}+\frac{\sigma_{n}^{2}}{M} \end{array}$$ (5)

As summation is a linear operator, so we can take the expectation inside to evaluate the expression

$$\begin{array}{c} E\left[P_{Y}\right]=\sum_{i=1}^{N}X^{2}E\left[\cos^{2}\left(\theta_{i}\right)\right]E\left[\alpha_{i}^{2}\right]+\sum_{i=1}^{N}\sum_{\begin{array}{c} j=1\\ j\neq i \end{array}}^{N}X^{2}E\left[\cos\left(\theta_{i}\right)\cos\left(\theta_{j}\right)\right]E\left[\alpha_{i}\alpha_{j}\right]+\frac{\sigma_{n}^{2}}{M} \end{array}$$ (6)

Since all *θ*_*i*_, *θ*_*j*_ *h*_*i*_ and *h*_*j*_ are i.i.d random therefore *E*[*θ*_*i*_]≈*E*[*θ*_*j*_]≈*E*[*θ*], *E*[*α*_*i*_]≈*E*[*α*_*j*_]≈*E*[*α*] and $E\left[\alpha_{i}^{2}\right]\approx E\left[\alpha^{2}\right]$. Also we know that $Var\left(\alpha \right)=\sigma_{\alpha }^{2}=\left(2-\frac{\pi }{2}\right)b^{2}$ and $E\left[\alpha \right]=\mu_{\alpha }=\left(\sqrt{\frac{\pi }{2}}\right)b$. So Eq (6) can be rewritten as follows

$$\begin{array}{c} E\left[P_{Y}\right]=NX^{2}E\left[\cos^{2}\left(\theta \right)\right]E\left[\alpha^{2}\right]+N\left(N-1\right)X^{2}E\left[\cos\left(\theta \right)\right]E\left[\cos\left(\theta \right)\right]E\left[\alpha \right]E\left[\alpha \right]+\frac{\sigma_{n}^{2}}{M} \end{array}$$ (7)

Now putting values from Eqs (35) and (36) (for derivation see Appendix) and values of *E*[*α*] and *E*[*α*^2^] in Eq (7) we get the following result

$$\begin{array}{c} E\left[P_{Y}\right]=Nb^{2}X^{2}\left[1+\frac{\sin(2\phi )}{2\phi }\right]+\frac{\pi N\left(N-1\right)b^{2}X^{2}}{2}{\left[\frac{\sin(\phi )}{\phi }\right]}^{2}+\frac{\sigma_{n}^{2}}{M} \end{array}$$ (8)

Here *ϕ* is the distribution limit over the phase error and *b* is the mode of Rayleigh fading. Notice in Eq (8) that, the noise effect is mitigated by the length of pseudo noise (PN) chip code. As the length of chip code increases, the effect of noise decreases and that is the magic of the spread spectrum system. Detailed analysis of results obtained by implementing Eq (8), is presented in energy consumption section.

### Average Probability of Error

To evaluate the bit error rate of the proposed system, let the transmitted signal is modulated using BPSK. The signal received at the base station is a combination of the signal sent as well as the added noise during propagation and both are identically independent random variables i.e, *Y* = *s* + *n*. The probability of error is given by the following expression

$$\begin{array}{c} P_{e}=0.5erfc\left(\frac{\mu_{Y}}{\sqrt{2\sigma_{Y}^{2}}}\right) \end{array}$$ (9)

Since *θ*_*i*_ and *α* are two different i.i.d random variables, therefore

$$\begin{array}{c} \mu_{Y}=\mu_{s}+\mu_{n} \end{array}$$ (10)

Putting values of *μ*_*s*_ and *μ*_*n*_ in Eq (10) and de-correlate with pseudo noise chipping code *c*_*j*_, we get

$$\begin{array}{c} \mu_{Y}=\frac{1}{M}E\left[\sum_{i=1}^{N}\sum_{j=1}^{M}\left(\alpha_{i}X_{i}c_{j}\cos\left(\theta_{i}\right)\right)\times c_{j}\right]+\frac{1}{M}E\left[\sum_{j=1}^{M}n\times c_{j}\right] \end{array}$$ (11)

In the first part of Eq (11), $1/M\sum_{j=1}^{M}c_{j}\times c_{j}=1$, whereas in the second part since *n* represents *AWGN* and its expectation results in 0, therefore the whole second part becomes zero and Eq (11) is reduce to the following form.

$$\begin{array}{c} \mu_{Y}=X\sum_{i=1}^{N}E\left[\alpha_{i}\right]E\left[\cos\left(\theta_{i}\right)\right] \end{array}$$ (12)

In Eq (12), *α*_*i*_, *α*_*j*_, *θ*_*i*_ and *θ*_*j*_ are identically distributed random process, therefore their expectation is approximately equal to their own-self without subscript. Putting values from Eq (35) (see appendix for derivation) and the expectation of *E*[*α*], Eq (12) can be reduced to the following form,

$$\begin{array}{c} \mu_{Y}=\left(\sqrt{\frac{\pi }{2}}\right)b\left(N\times X\right)\frac{\sin(\phi )}{\phi } \end{array}$$ (13)

Here *ϕ* is the limit on the distribution of phase error and *b* is the mode of Rayleigh fading *α*_*i*_, a zero mean Gaussian distributed random process.

Since *Y* is the sum of two i.i.d random variables, so its variance can be calculated as follow

$$\begin{array}{c} \sigma_{Y}^{2}=\sigma_{s}^{2}+\sigma_{n}^{2} \end{array}$$ (14)

putting values of the de-correlated signal and noise variance in Eq (14). We know that noise *n* and chipping code *c*_*j*_ are independent of each other and variance of *AWGN* noise correlated with *PN* sequence summed over *M* is $\sigma_{n}^{2}/M$, where $\sigma_{n}^{2}=N_{0}/2$ to distinguish it from Eq (14). We also know that *α*_*i*_ and *θ*_*i*_ are independent random processes so *α*_*i*_ ≈ *α* and *θ*_*i*_ ≈ *θ*, so the above equation will take the following form,

$$\begin{array}{ccc} \sigma_{Y}^{2} & = & Var\left[\sum_{i=1}^{N}\alpha_{i}X\cos\left(\theta_{i}\right)\right]+Var\left[\sum_{j=1}^{M}n\times c_{j}\right]\\ & = & \sum_{i=1}^{N}Var\left[\alpha_{i}X\cos\left(\theta_{i}\right)\right]+\frac{N_{0}}{2M} \end{array}$$ (15)

Eq (15) shows that the effect of noise is considerably mitigated by the *PN* sequence. As the length *M* of *PN* sequence increases the effect of noise decreases which means that the noise is evenly distributed over all the communicating nodes instead of just one or two.

Now taking values from Eq (39) (see appendix for derivation), Eq (15) will become

$$\begin{array}{c} \sigma_{Y}^{2}=NX^{2}b^{2}\left[1-\frac{\pi }{2}\left(\frac{\sin(\phi )}{\phi }\right)+\frac{\sin(2\phi )}{2\phi }\right]+\frac{N_{0}}{2M} \end{array}$$ (16)

Now by putting values from Eq (13) and (16), in Eq (9) we get the following relation

$$\begin{array}{c} P_{e}=0.5erfc\left(\frac{\left(\sqrt{\frac{\pi }{2}}\right)b\left(N\times X\right)\frac{\sin(\phi )}{\phi }}{\sqrt{2(NX^{2}b^{2}\left[1-\frac{\pi }{2}\left(\frac{\sin(\phi )}{\phi }\right)+\frac{\sin(2\phi )}{2\phi }\right]+\frac{N_{0}}{2M})}}\right) \end{array}$$ (17)

Eq (17) can be represented in form of *SNR* with some further simple manipulation. As *X*^2^ = *E*_*b*_, the probability of error relation in Eq (17) can be rewritten in the following form

$$\begin{array}{c} P_{e}=0.5erfc\left(\sqrt{\frac{\pi }{2}}\left(\frac{\sin(\phi )}{\phi }\right)b\sqrt{\frac{N^{2}\left(E_{b}/N_{0}\right)}{\left(2Nb^{2}\left[1-\frac{\pi }{2}{\left(\frac{\sin(\phi )}{\phi }\right)}^{2}+\frac{\sin(2\phi )}{2\phi }\right]\left(\frac{E_{b}}{N_{0}}\right)+\frac{1}{M}\right)}}\right) \end{array}$$ (18)

Special Case: reduction in transmit power

Spread spectrum technique allow nodes to transmit at a low power which extends the battery time of each node. This extension in battery time is further improved by collaborative communication by reducing the transmit power by a factor of *N*, where *N* is the number of transmitter nodes. To prove this let the amplitude of signal transmitted by each collaborative node is $X=\pm \sqrt{E_{b}}/N$, then the expression in Eq (18) can be expressed as follows.

$$\begin{array}{c} P_{e}=0.5erfc\left(\sqrt{\frac{\pi }{2}}\left(\frac{\sin(\phi )}{\phi }\right)b\sqrt{\frac{\left(E_{b}/N_{0}\right)}{\left(\frac{2b^{2}}{N}\left[1-\frac{\pi }{2}{\left(\frac{\sin(\phi )}{\phi }\right)}^{2}+\frac{\sin(2\phi )}{2\phi }\right]\left(\frac{E_{b}}{N_{0}}\right)+\frac{1}{M}\right)}}\right) \end{array}$$ (19)

It is clear from Eq (19) that the transmit power is reduced by factor of *N* due to the use of collaborative communication. It is also interesting to see that the length *M* of *PN* sequence(chip code) also contributes to this reduction in power. The energy efficiency of the proposed system can be analyzed against different number of collaborative nodes as well as for different values of *BER*.

## Energy efficiency of collaborative communication in WSN

Energy consumption is an important figure of merit in evaluating performance of wireless sensor networks. In order to analyzed the energy efficiency of collaborative communication this section presents energy consumption models for *SISO* and collaborative communication. Both the models are derived and analyzed for the sack of comparison and evaluating performance of the collaborative communication. This theoretical models reflect the effect on the energy consumption when collaborative communication is used in combination with the spread spectrum approach. These models have been used to compute break-even-distances, and an analysis of both the models has been presented in the results and discussion section to analyze the effect of both systems on coverage range.

### *SISO* energy consumption model

Energy consumption of a communication system is typically the combined energy consumed by the transmitter (*P*_*tr*_) and receiver (*P*_*rv*_). therefore energy consumption over a single bit in *SISO* can be represented by the following relation

$$\begin{array}{c} E_{SISO}=\frac{P_{tr}+P_{rv}}{C_{i}\times R_{s}} \end{array}$$ (20)

where *R*_*s*_ is the bit transmission rate and *C*_*i*_ is the PN sequence.

In this case, a simplified path loss model be applied to calculate the desired transmission power as argued in [41]. Consider *G*_*t*_ and *G*_*r*_ be the gain of transmitter and receiver antenna respectively, and if *G*_*t*_ = *G*_*r*_ = 1, then the total energy consumption of the transmitter *P*_*tr*_ is given by the following relation [42].

$$\begin{array}{c} P_{tr}=P_{cir}+\frac{{\left(4\pi \right)}^{2}P_{r}d^{\beta }}{d_{r}^{\beta -2}\lambda^{2}} \end{array}$$ (21)

power required for transmitter circuit operation is represented by *P*_*cir*_, power of received signal *P*_*r*_, *λ* = *c*/*f*_0_, where c represents speed of light, *f*_0_ represents carrier frequency, *β* represents exponent of path loss, *d* represents distance between receiver and transmitter, *d*_*r*_ represents reference distance for far-field region.

The minimum required received power *P*_*r*_, to achieve the desired bit error rate can be calculated as follow

$$\begin{array}{c} P_{r}=P_{s}+r_{eber} \end{array}$$ (22)

*P*_*s*_ represents receiver’s sensitivity(in Watt), required to obtain required BER in noisy(AWGN) channel only, and *r*_*eber*_ is *E*_*b*_/*N*_0_(in Watt) used to obtain desired BER for a Rayleigh faded AWGN system. *r*_*eber*_ in [38], is shown to be calculated as

$$\begin{array}{c} r_{eber}=\frac{\left({\left(1-2P_{e}\right)}^{2}/1-{\left(1-2P_{e}\right)}^{2}\right)}{{\left(erfc^{-1}\left(2P_{e}\right)\right)}^{2}} \end{array}$$ (23)

*erfc*^−1^ is inverse of complimentary error function $erfc\left(x\right)=\frac{2}{\sqrt{\pi }}\int_{x}^{+\infty }e^{-t^{2}}dt$.

Taking values from Eqs (21)–(23), total consumption of energy for SISO systems can be achieved as

$$\begin{array}{c} E_{SISO}=\left(P_{cir}+\frac{{\left(4\pi \right)}^{2}P_{s}r_{eber}d^{\beta }}{dr^{\beta -2}\lambda^{2}}+P_{rv}\right)/C_{i}\times R_{s} \end{array}$$ (24)

### Collaborative communication energy consumption model

Energy consumption in SISO model is given by the sum of energy consumed in node-to-node communication *E*_*l*_ (local communication) and energy consumed by communicating with base station *E*_*t*_. Energy consumption of both local communication and communication with the base station may be represented as follow

$$\begin{array}{c} E_{COL}=E_{l}+E_{t} \end{array}$$ (25)

Both channels; channel among collaborative nodes and channel between a collaborative node and base station are Rayleigh fading channels. The distance among collaborative nodes is considered to be maximum (local communication) leading to maximum energy consumption, although distance of collaborative nodes from base station may vary. Energy consumption in case of local communication can be represented as follow

$$\begin{array}{c} E_{l}=\frac{P_{tr\_l}+NP_{rv\_l}}{R_{s}} \end{array}$$ (26)

Here *N* is the number of collaborative nodes in the sensor network. *P*_*tr*_*l*_ can be derived from Eq (21), can be written in the following form

$$\begin{array}{c} E_{l}=(P_{cir}+\frac{{\left(4\pi \right)}^{2}P_{s}r_{rber\_l}d_{l}^{\beta }}{d_{l}^{\beta -2}\lambda^{2}}+NP_{rv\_l})/R_{s} \end{array}$$ (27)

Energy consumed during communication between collaborative nodes and base station is given by

$$\begin{array}{c} E_{t}=\frac{P_{tr\_t}+P_{rv}}{c_{i}\times R_{s}} \end{array}$$ (28)

where *P*_*tr*_*t*_ is total energy, which is consumed by all (*N*) collaborative nodes and can be rewritten as

$$\begin{array}{c} P_{tr\_t}=NP_{cir}+\frac{{\left(4\pi \right)}^{2}P_{r\_t}d^{\beta }}{Ndr^{\beta -2}\lambda^{2}} \end{array}$$ (29)

To obtain the desired Bit Error Rate(BER), the minimum required received power *P*_*r*_*t*_ may be written as

$$\begin{array}{c} P_{r\_t}=P_{S}+r_{col\_ber} \end{array}$$ (30)

where *r*_*col*_*ber*_ represents ratio between *E*_*b*_/*N*_0_ (for system with phase error), AWGN and Rayleigh fading, and For systems with AWGN only, this ratio is equal to *E*_*b*_/*N*_0_ (in Watt), to achieve the desired BER. *r*_*col*_*ber*_ can be re-written as

$$\begin{array}{c} r_{col\_ber}=\frac{BER^{-1}\left(P_{e},N\right)}{{\left(erfc^{-1}\left(2P_{e}\right)\right)}^{2}} \end{array}$$ (31)

here *BER*^−1^ is the inverse function of Eq (19). Therefor *E*_*t*_ can be written as follow

$$\begin{array}{c} E_{t}=(NP_{cir}+\frac{{\left(4\pi \right)}^{2}P_{r\_t}d^{\beta }}{Nd_{r}^{\beta -2}\lambda^{2}}+P_{rv})/c_{i}\times R_{s} \end{array}$$ (32)

Taking values from Eqs (27) and (32), to represent total energy consumption of collaborative communication as follow

$$\begin{array}{c} E_{COL}=\frac{1}{R_{s}}\left(\left(P_{cir}+\frac{{\left(4\pi \right)}^{2}P_{s}r_{rber\_l}d_{l}^{\beta }}{d_{l}^{\beta -2}\lambda^{2}}+NP_{rv}\right)+\left(\frac{NP_{cir}+\frac{{\left(4\pi \right)}^{2}P_{r\_t}d^{\beta }}{Nd_{r}^{\beta -2}\lambda^{2}}+P_{rv}}{c_{i}}\right)\right) \end{array}$$ (33)

The total energy saving for the proposed collaborative communication model can be achieved using the following equation.

$$\begin{array}{c} E_{saving}\left(\%\right)=\left(\frac{E_{SISO}-E_{COL}}{E_{SISO}}\times 100\right)\%. \end{array}$$ (34)

Energy saving for small distances is dominated by circuit energy consumption. Saving is 0 in case where *E*_*SISO*_ = *E*_*COL*_, and the distance in this case is known as “break-even-distance”.

## Results and Discussion

**Fig 3:**
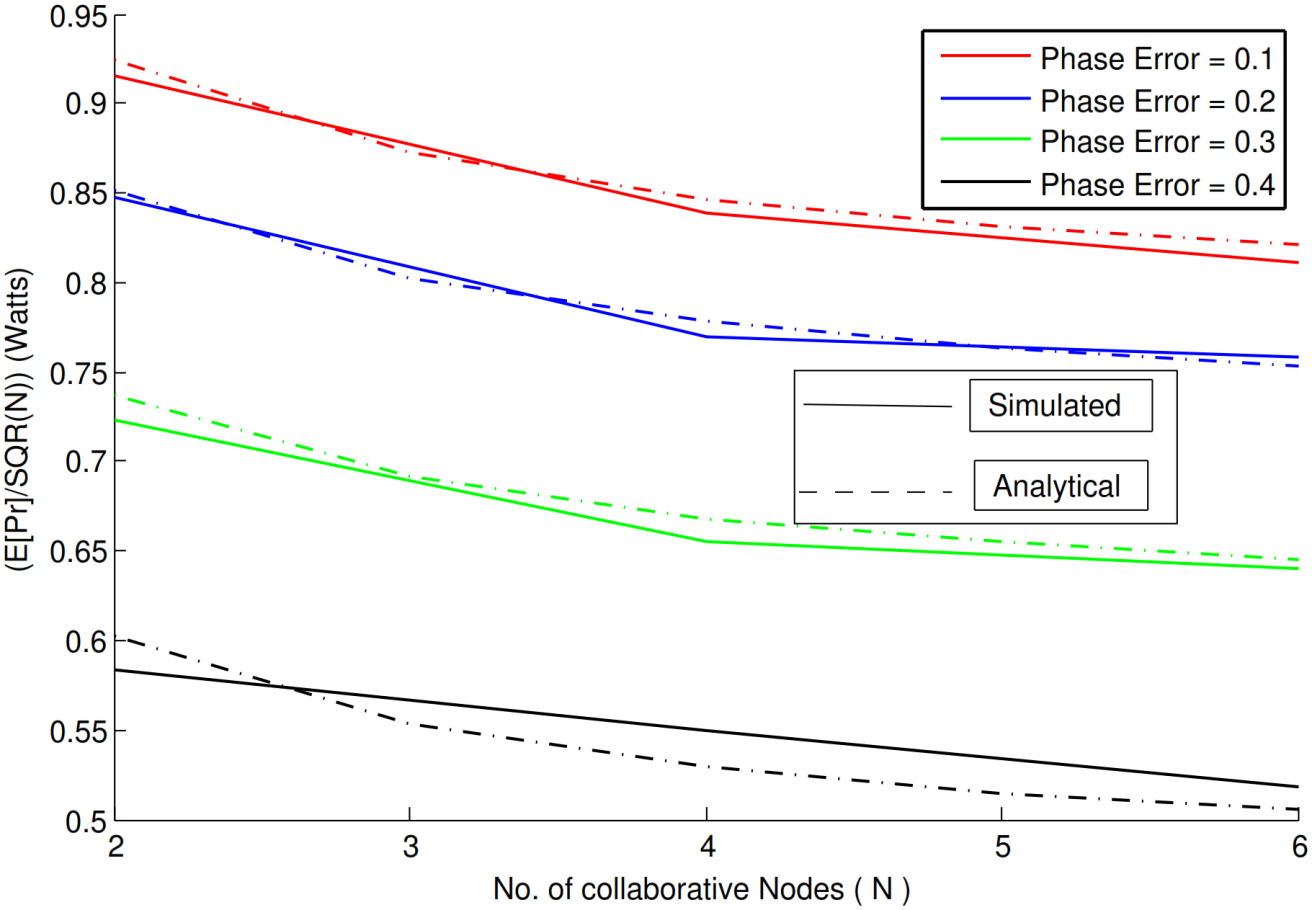
Normalized average received power vs. number of collaborative nodes with Rayleigh fading.

This section is devoted to analyzed the behavior of the proposed system. For this purposed results obtained from analytical and simulated experiments are compared shown in the following series of figures. The comparison shows that the theoretical and simulation are a perfect match. Both analytical and simulated results are obtained using *Monte Carlo* simulation. For experimental purposes, the phase error is assumed to be uniformly distributed over a range from −*ϕ* to *ϕ*. Four different ranges of phase values has been used for the sack of experimental analysis i.e, {−0.1*π* to 0.1*π*}, {−0.2*π* to 0.2*π*}, {−0.3*π* to 0.3*π*} and {−0.4*π* to 0.4*π*}. 46-bit Hadamard codes are used as PN sequences to spread and de-spread the signal at the sender and receiver. It has been observed that the use of these codes (spread spectrum), reduces the effect of noise, thereby improving not only the received power but also the bit error rate(BER). Results for normalized received power are shown in Fig 3.

**Fig 4:**
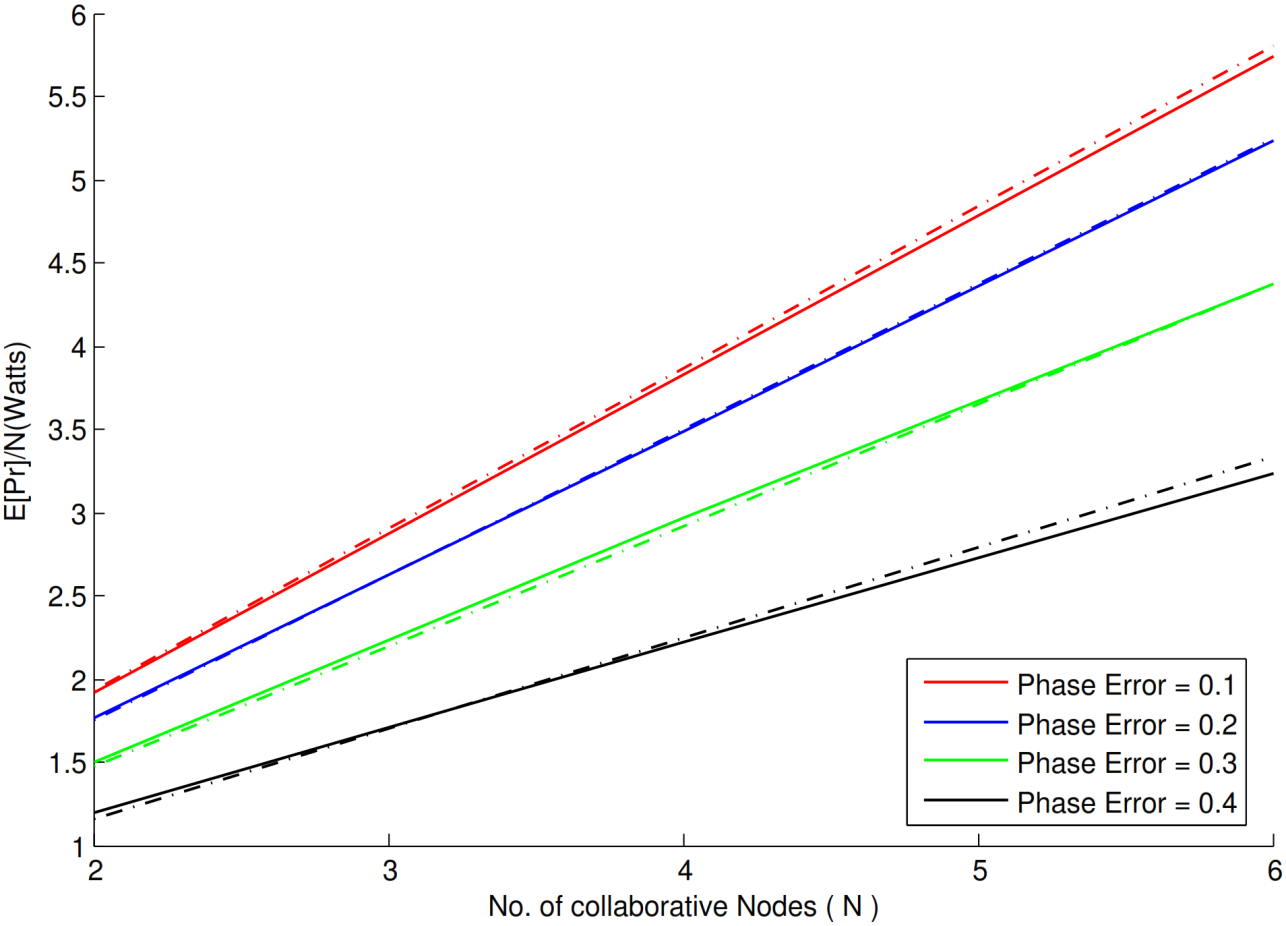
Average received power/N vs. number of collaborative nodes with Rayleigh fading.

It is clear from Fig 3 that increase in phase error has inverse effect on the normalized received power. As the phase error increases, the normalized received power decreases. A closer look at the figure reveals that 9-10% approximate decrease occured for the phase error over the interval {−0.1*π* to 0.1*π*}, 17-18% for the phase error over the interval {−0.2*π* to 0.2*π*}. The error seems to increase with increase in phase error i.e, the interval over {−0.3*π* to 0.3*π*} produces an approximate decrease of 31-32% whereas the interval over {−0.4*π* to 0.4*π*} produces an approximate of 47-48% decrease in the normalized received power in comparison to the *N*^2^ received power without phase errors. Another comparison between simulated and analytical results is shown in Fig 4, based on the total average received power i.e, *“power/N”* in the presence of fading.

It is clear from the figure linear increase occurs in the total received power with an increase in the number of collaborative nodes. Therefore it safe to conclude that in collaborative communication, the number of collaborative nodes have a impact on the total received power. Even in the presence of phase error and fading a significant gain in the received power is recorded. It proves that collaborative communication in combination with spread spectrum not only can mitigate the fading effect but also the noise to achieve such gain in the received power. Fig 4 shows an approximate of 0.65 − 0.66*N*^2^ gain in the received power over the phase error interval of {−0.3*π* to 0.3*π*}. In case of interval over {−0.4*π* to 0.4*π*}, there seems to be a negative impact on the gain in received power, but still it resulted in a gain of 0.51 − 0.52*N*^2^.

**Fig 5:**
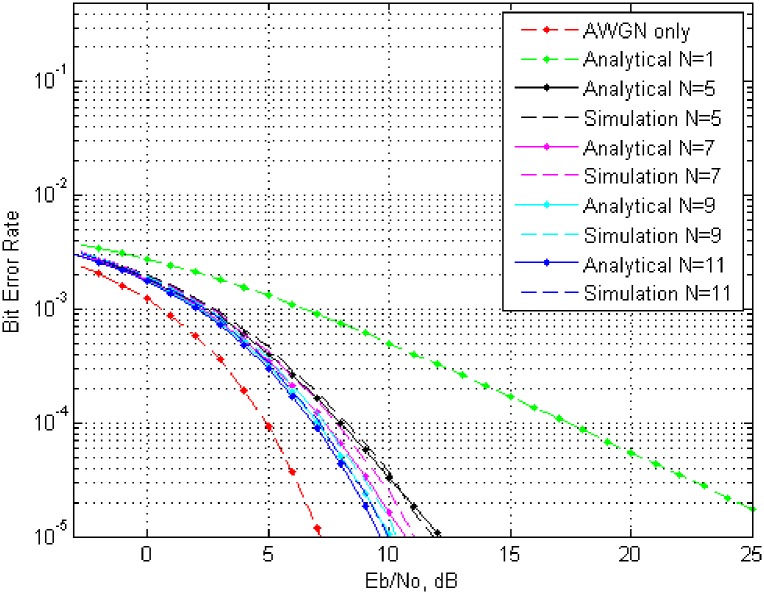
*BER* over interval {−0.1*π* to 0.1*π*} for different number of nodes with fading and total transmitted energy *E*_*b*_/*N*_0_.

**Fig 6:**
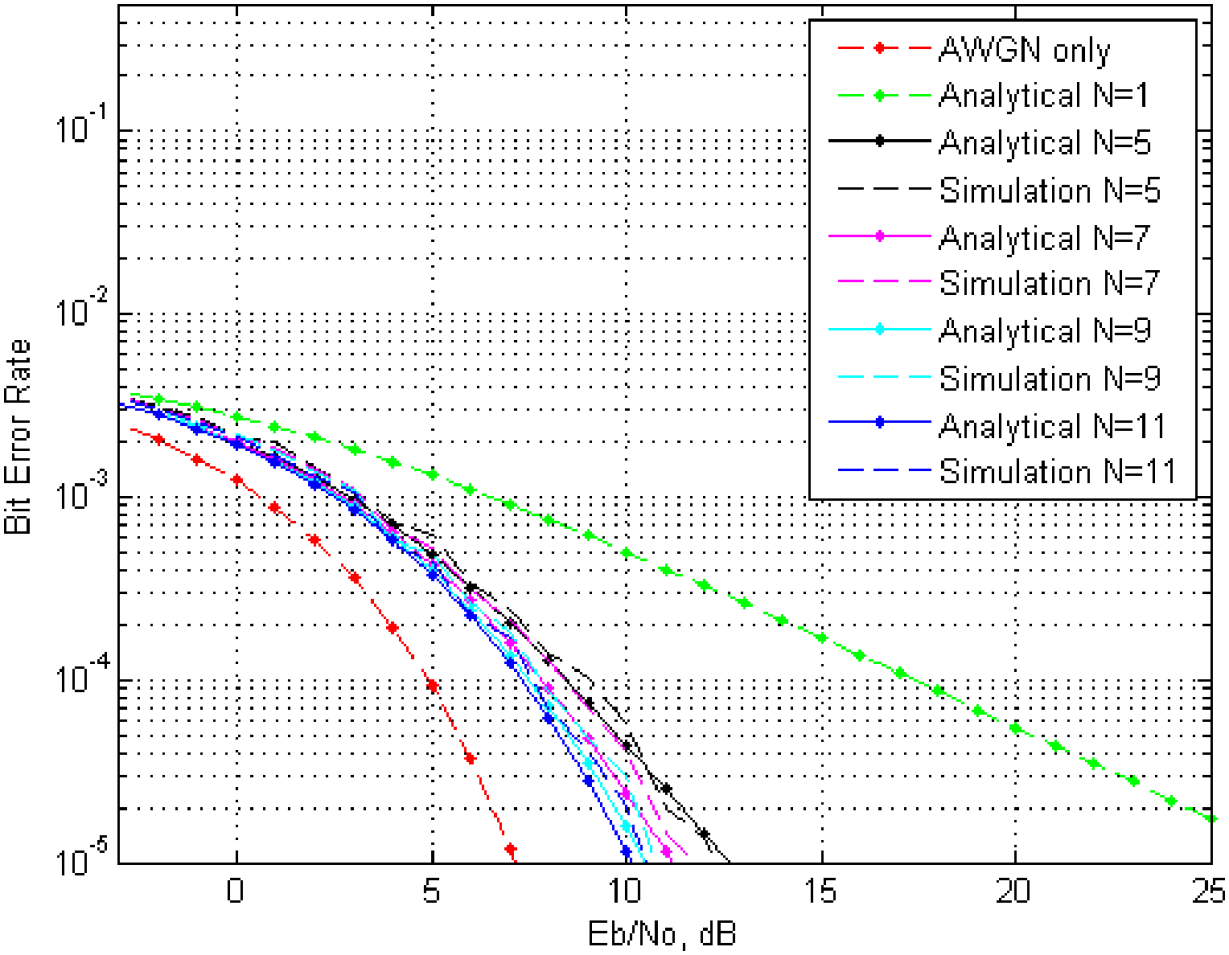
*BER* over interval {−0.2*π* to 0.2*π*} for different number of nodes with fading and total transmitted energy *E*_*b*_/*N*_0_.

Now to analyze bit error rate, let the energy consumed by one bit is represented by *E*_*b*_ = *N*^2^, then the energy consumed by all collaborative nodes must be *E*_*b*_ = *N*. Analytical results based on the calculation from Eq (19) versus the simulated results are plotted in Figs 5 and 6. For the sack of space limitation, two of four results are included for phase error over the interval {−0.1*π* to 0.1*π*} and {−0.2*π* to 0.2*π*}, but they should be enough to reflect the trend of *BER* with Rayleigh fading and *AWGN* for a varied number of collaborative node.

Further analysis of the *BER* curves reveal the effect of number of collaborative nodes. From Fig 5, with phase error interval over {−0.1*π* to 0.1*π*}, it is clear to achieve a *BER* of 10^−4^ in case of *AWGN* only, a power of approximately 5*dB* is required with no fading, whereas in case of a single node with fading only, a power of 17*dB* is required. By increasing the number of node from one to five with fading, the required power to achieve a *BER* of 10^−4^, is approximately 8*dB*. Similarly the required power reduces to approximately 8*dB*, 7.5*dB* and 7.2*dB* for a number of 7,9 and 11 collaborative nodes respectively. Fig 5 in case of phase error over {−0.2*π* to 0.2*π*}, The required power in case *AWGN* only is same but there is a slight raise in the remaining cases like fading only requires a power of 17.6*dB*, for *N* = 5 it is 9.2*dB* for *N* = 7 it is 8*dB*, for *N* = 9 it is approximately 7.7*dB* and for *N* = 11 it is 7.5*dB*. Here it can be seen that an increase in number of collaborative nodes *N*, reduces the desired power requirement whereas an increase in the phase error has inverse effect over the required power. Similar trends has been observed for phase error interval over {−0.3*π* to 0.3*π*} and {−0.4*π* to 0.4*π*}.

As mentioned above, it is clear from Figs 3 and 4 that the gain in received power raises as the number of collaborative nodes *N* increases, but increasing the number of node also mean an increase in power consumption due to circuit operations. To perform an analysis of the energy cosumed by the proposed approach, break-even distances are measured over different phase error intervals and different number of collaborative nodes *(N)*. Parameters of the off-the-shelf products, like *CC2420* and *AT86RF21* are considered for this analysis. The maximum distance between any two nodes is considered to 1*m*, accepted value of *BER* for energy consumption analysis is 10^−5^ whereas the path loss exponent *β* ranges from 4.0–6.0 [43]. A detailed summary of product information about off-the-shelf products i.e, *CC2420* and *AT86RF21*, is given in Table 1.

**Fig 7:**
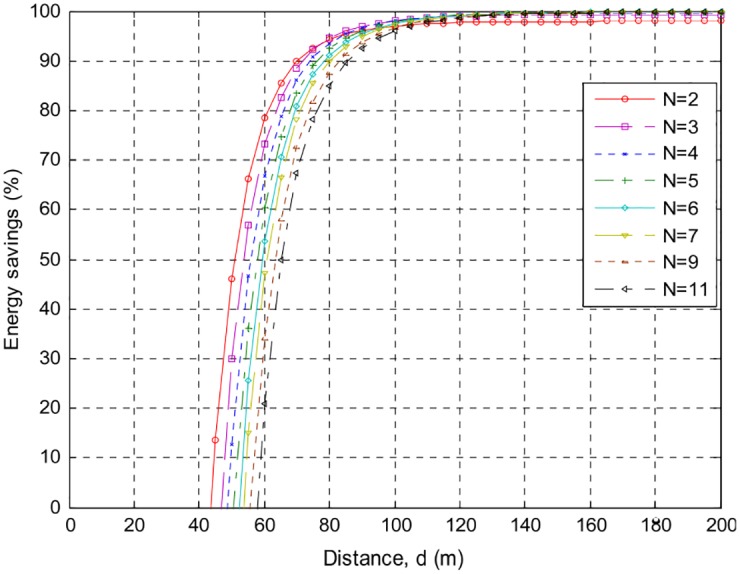
Percentage energy savings and break-even distances over {−0.1*π* to 0.1*π*}, for AT86RF212, with different number of nodes.

**Fig 8:**
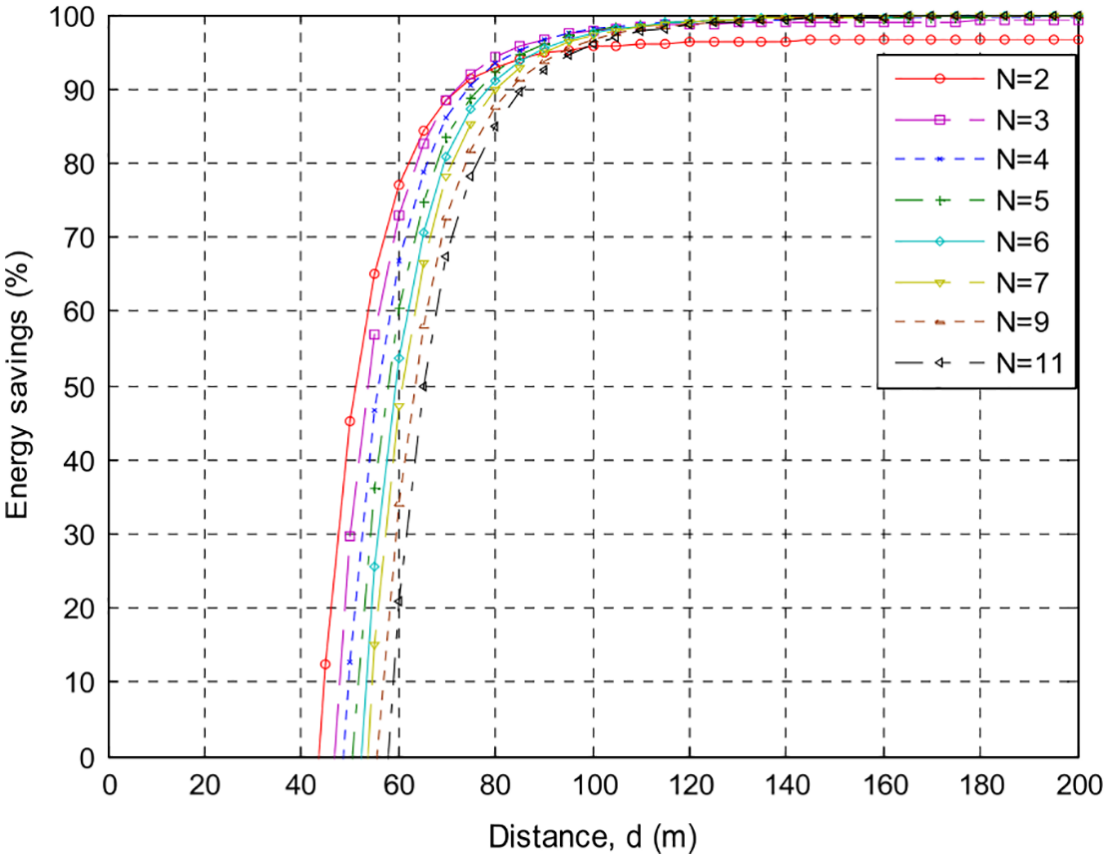
Percentage energy savings and break-even distances over {−0.3*π* to 0.3*π*} for AT86RF212, with different number of nodes.

**Fig 9:**
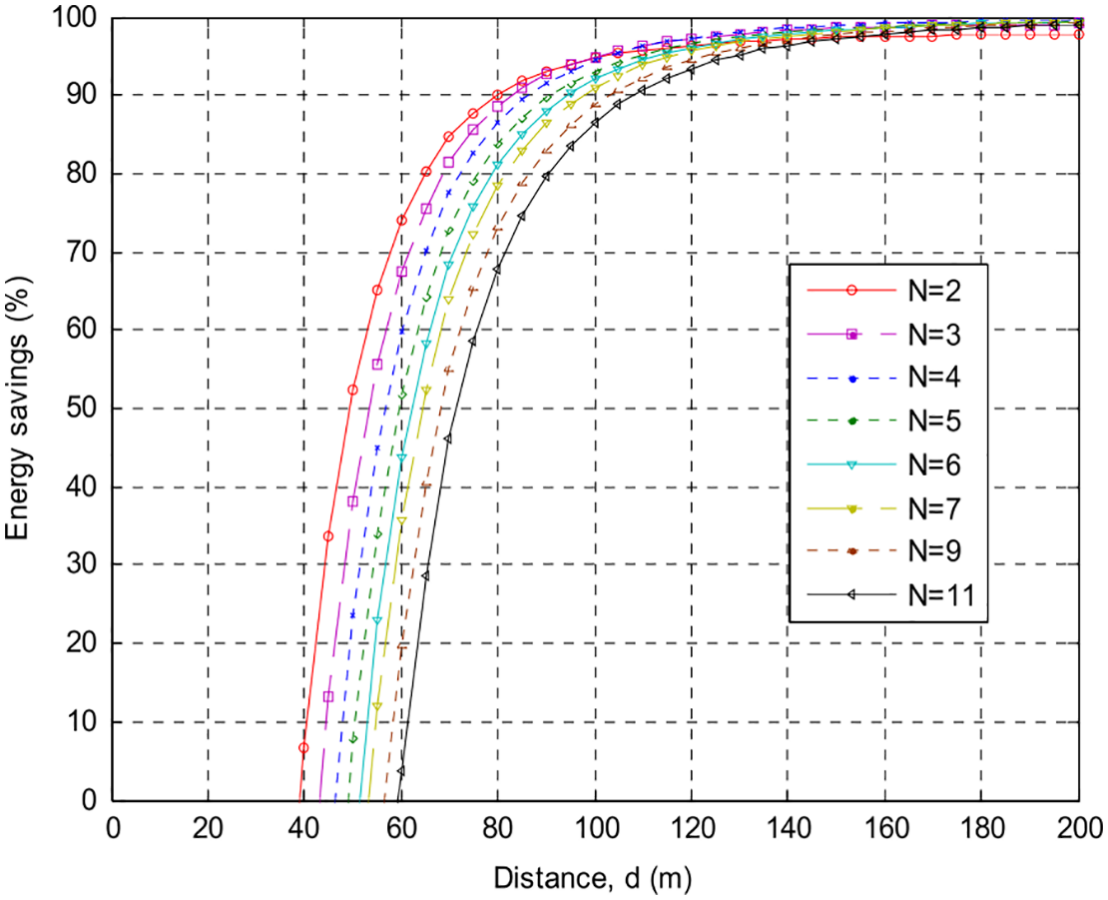
Percentage energy savings and break-even distance over {−0.1*π* to 0.1*π*} for CC2420, with different number of nodes.

**Fig 10:**
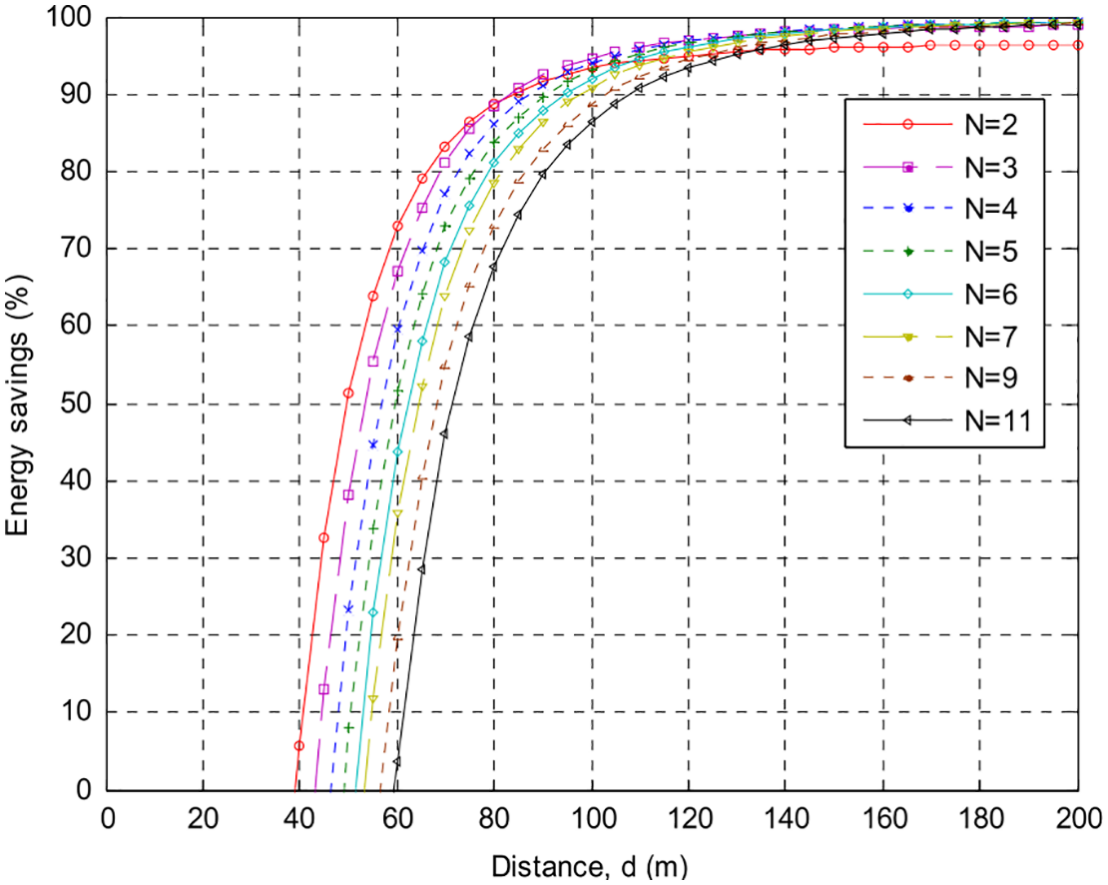
Percentage energy savings and break-even distance ove {−0.3*π* to 0.3*π*} for CC2420, with different number of nodes.

The following Figs 7–10 present the relation between percentage energy savings with the number of collaborative nodes *(N)*. It can be observed that an increase in the number of collaborative nodes results in a raise in break-even distances. For the sack of analysis break-even distances for *AT86RF212* and *CC2420* are presented over different phase error intervals. *CC2420* has long break-even distance but less energy efficient than *AT86RF212* both at 100*m* and 200*m* distances. It can be seen that *AT*86*RF*212 has fast energy saving convergence than *CC*2420 after reaching the break-even distances showing that *AT*86*RF*212 more stable than *CC*2420. A more closer look shows that *AT*86*RF*212 stabilizes at approximately 117*m* whereas *CC*2420 gets stabilize at approximately 146*m*.

Table 2 shows a detailed summary of Break-even distances for both *CC2420* and *AT86RF212*. It is analyzed that an increase in the distances causes increase in the energy preservation of collaborative communication and becomes constantly steady after a specific distance. Percentage energy preservation for both the products over different phase error intervals for distances of *100m* and *200m* are shown in Tables 3 and 4.

**Table 2:** Break-even distance for CC2420 and AT86RF212.

N	Break-even distance CC2420(m)	Break-even distance AT86RF212(m)
2	40.5	44.5
3	43.7	46.7
4	47.6	50.5
5	50.2	52.1
6	52.5	54
7	54.7	54.7
9	58.1	57.1
10	61.1	59.5

**Table 3:** Percentage energy preservation based on the parameters of CC2420.

N	phase error 0.1*π*		phase error 0.2*π*		phase error 0.3*π*		phase error 0.4*π*	
	200m	100m	200m	100m	200m	100m	200m	100m
2	98	95.5	97.5	94.9	97	94.3	95	92
3	99.4	95.7	99.5	95.5	98.3	95.5	99	94.6
4	99.8	94.8	99.7	94.4	99.5	94.3	99.4	94.5
5	99.9	93.5	99.8	93.3	99.6	93.3	99.5	93.5
6	99.6	92.4	99.6	92.5	99.6	92.3	99.5	92.4
7	99.6	91.5	99.5	91.4	99.5	91.3	99.5	91.5
9	99.5	88.4	99.5	88.4	99.5	90.5	99.5	89.4
11	99.2	86.8	99	86.5	98.6	89.5	98.5	87

**Table 4:** Percentage energy preservation based on the parameters of AT86RF212.

N	phase error 0.1*π*		phase error 0.2*π*		phase error 0.3*π*		phase error 0.4*π*	
	200m	100m	200m	100m	200m	100m	200m	100m
2	98.4	97.4	98	95	97	95.9	95.5	94.5
3	99.8	98.4	99.5	95.3	98.5	98.3	99.1	97.8
4	99.8	98.1	99.9	94.3	98.6	98.4	99.6	97.9
5	100	97.8	100	93.3	97.9	98	99.9	97.8
6	100	97.6	100.1	92.3	97.8	97.9	99.97	97.5
7	100.1	97.5	100.1	91.3	97.6	97.6	100.2	94.4
9	100	96.7	100.1	89.2	96.7	96.7	100	96.2
11	100.2	96.3	99.3	86.4	96.3	96.3	99.1	96.8

### Comparison with other Approaches

Energy preservation efficiency measurement technique can be classified as one of Multihop, Cooperative communication or beamforming [5, 11, 12, 44]. The proposed system comes under the category of beamforming, since beamforing focuses multiple signals on a single point to gain different advantages [9]. collaborative communication follow identical approach as beamforming, since in collaborative communication there is a single receiver known as the base station. So collaborative communication can be considered as an extension of beamforming and both can be used in combination to achieve different goals like high gain in received power.

#### Comparison with Multihop communication

In this type of communication the sending device is unable to transmit information directly to a base station, instead it forward the information to an intermediate node in the direction toward base station, which in turn forward it either to the base station if in range or forward it in the same fasion to another neighbor that is closer to the base station [5, 45]. It is an impressive approach capable of not only significant improvements in signal reception but also tremendously reduces the transmit power of the sender to preserve its energy.

**Fig 11:**
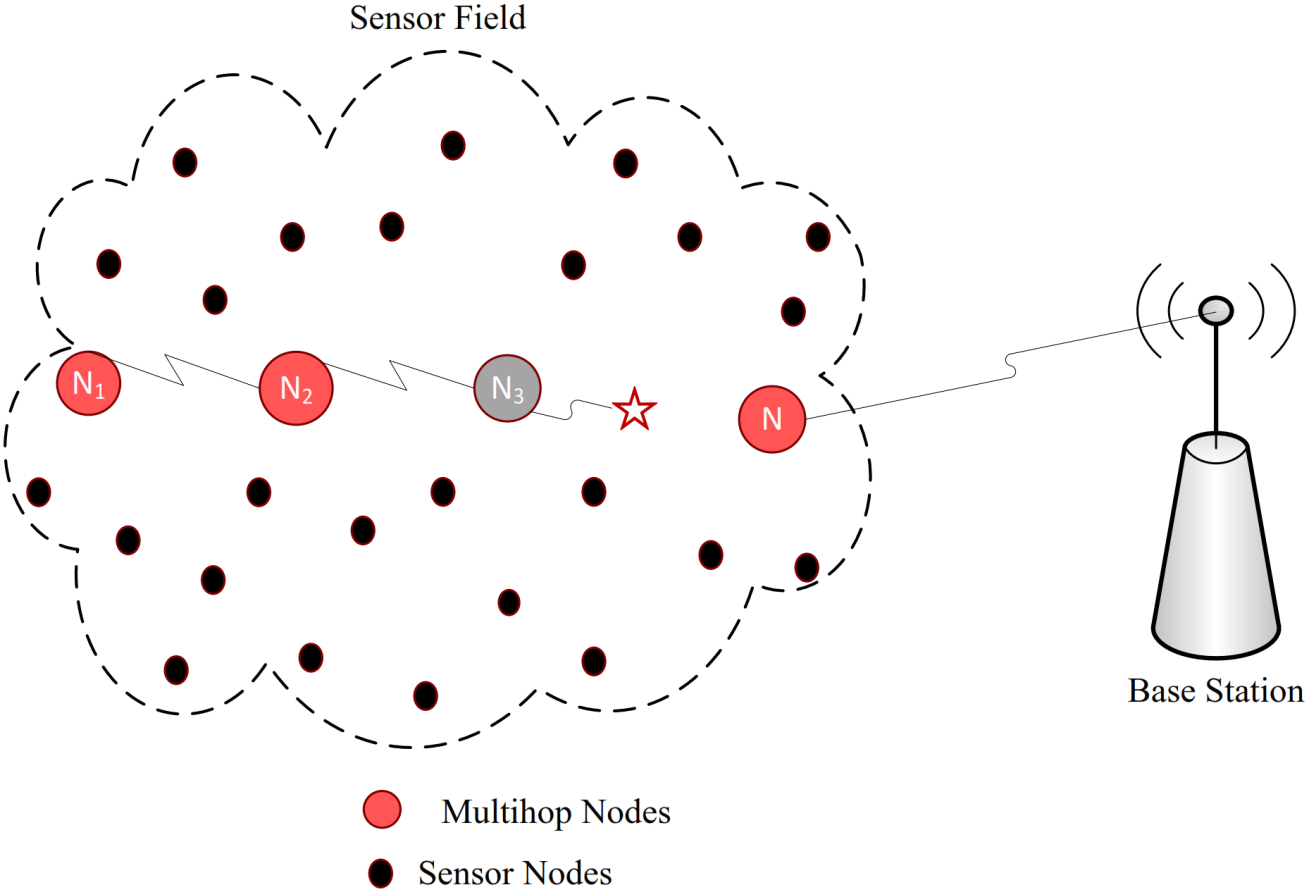
Multihop system in case of node failure.

However due to the involvement of multiple intermediate nodes, this approach has the tendency to of becoming more complex as the number of intermediate nodes increases from source to the base station. Additionally it has worse effect in terms of energy consumption on all nodes from source to base station in case of a retransmission. In terms of range, multihop communication can transmit over long distances but suffer severely in case a node dies in the multihop path as shown in Fig 11, resulting in a sudden exponential growth in *BER*. Let consider a four nodes (*N* = 4) multihop path with each node having a range of 1*m*. In a multihop scenario these four nodes can transmit up to 16 (*N*× transmission power of a node) meters. Anything further will lead to the inclusion of new hop thereby increasing complexity as well as the overhead in case of retransmission. For the same number of nodes, collaborative communication can transmit up to 64 meters, as the output power is square of the input power from each node.

**Fig 12:**
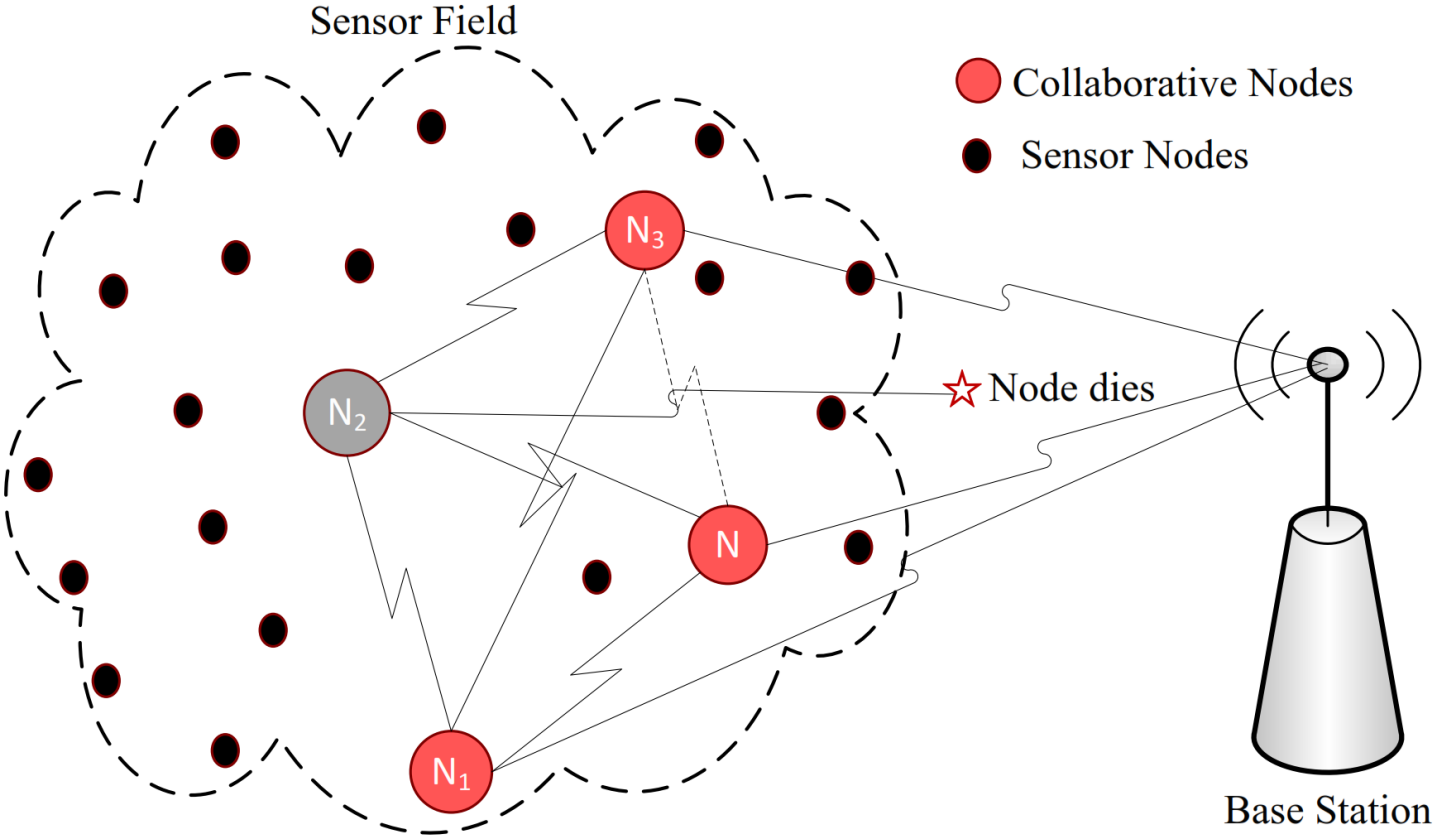
collaborative communication in case of node failure.

On the other hand in case of a node’s death, transmission in collaborative communication will not be effected, however *BER* may slightly increase as shown in Fig 12.

#### Comparison with Cooperative communication

In this approach a node cooperatively forwards any information received from its next neighbors to fulfill the requirement of cooperation [4]. It overcome the problem of node’s death by involving multiple next hop neighbors (redundancy). In other words cooperative communication can be thought of as multipath multihop communications, inheriting advantages of multihop communication as well as its problem in even more severe nature. However for the same amount of information transfer, energy source of many more nodes may effected as compared to multihop and collaborative communication. A node has to forward data it receives even if it makes no difference in the overall communication. The more dense the node deployment, the more severe the effect of energy draining for the same amount of information as compared to collaborative communication.

## Conclusion

A spread spectrum based energy efficiency mechanism using collaborative has been derived and analyzed in this article. The analysis is performed through a comparison between analytical and simulated results using received power, bit error rate and energy consumption as figures of merit. It has been proved that even if the received signals are unsynchronized in phase, using collaborative communication a significant power gain and well as better *BER* rates can be achieved. It has been observed in the analysis that *BER* is function of SNR whereas received power is a function of the number of collaborative nodes. For analysis of energy consumption the proposed system is compared with its counterpart *SISO* system and it has been observed that the performance of *SISO* is better at short distance whereas collaborative communication performs well at medium and large distances. To conclude it can be argued that although an increase in the number of collaborative node offers many benefits but the this number should be kept moderate so that the power consumption of circuit operation be kept from overwhelming the overall energy consumption of the system. The same approach can be extended to perform the same analysis in case of imperfect frequency as well as frequency and phase synchronization for collaborative communication systems.

## Mean values of trigonometric functions

### Mean value of cos(*θ*_*f*_)

$$\begin{array}{ccc} E[\cos\left(\theta_{f}\right)] & = & \int_{-\infty }^{\infty }\cos\left(\theta_{f}\right)P\left(\theta_{f}\right)d\left(\theta_{f}\right)\\ & = & \int_{-\phi }^{\phi }\cos\left(\theta_{f}\right)\frac{1}{2\phi }d\left(\theta_{f}\right)\\ & = & \frac{1}{2\phi }\int_{-\phi }^{\phi }\cos\left(\theta_{f}\right)d\left(\theta_{f}\right)\\ & = & \frac{\sin(\phi )}{\phi } \end{array}$$ (35)

### Mean value of cos^2^(*θ*_*f*_)

$$\begin{array}{ccc} E[\cos^{2}\left(\theta_{f}\right)] & = & \int_{-\infty }^{\infty }\cos^{2}\left(\theta_{f}\right)P\left(\theta_{f}\right)d\left(\theta_{f}\right)\\ & = & \int_{-\phi }^{\phi }\cos^{2}\left(\theta_{f}\right)\frac{1}{2\phi }d\left(\theta_{f}\right)\\ & = & \frac{1}{2\phi }\int_{-\phi }^{\phi }\cos^{2}\left(\theta_{f}\right)d\left(\theta_{f}\right)\\ & = & \frac{1}{2\phi }\left(\phi +\frac{\sin(2\phi )}{2}\right)\\ & = & \frac{1}{2}+\frac{\sin(2\phi )}{4\phi } \end{array}$$ (36)

### Variance of cos(*θ*_*f*_)

$$\begin{array}{c} Var\left(\cos\left(\theta_{f}\right)\right)=E\left[\cos^{2}\left(\theta_{f}\right)\right]-{\left(E\left[\cos\left(\theta_{f}\right)\right]\right)}^{2} \end{array}$$

Using Eqs (35) and (36) in the above equation we get

$$\begin{array}{ccc} Var(\cos\left(\theta_{f}\right)) & = & \frac{\sin(\phi )}{\phi }-\left(\frac{1}{2}+\frac{\sin(2\phi )}{4\phi }\right) \end{array}$$ (37)

### Variance of *hS* cos(*θ*_*f*_)

since all of these are independent random variables, therefore the multiplication can be calculated as

$$\begin{array}{ccc} Var[hS\cos\left(\theta_{f}\right)] & = & S^{2}[Var\left[h\right]{\left(E\left[\cos\left(\theta_{f}\right)\right]\right)}^{2}+{\left(E[h]\right)}^{2}Var\left[\cos\left(\theta_{f}\right)\right]\\ & & +\;Var\left[h\right]Var[\cos\left(\theta_{f}\right)]] \end{array}$$ (38)

Since we know from Eqs (35) and (37) that, $Var\left(h\right)=\sigma_{h}^{2}=\left(2-\frac{\pi }{2}\right)b^{2}$ and $E\left[h\right]=\mu_{h}=\left(\sqrt{\frac{\pi }{2}}\right)b$ by putting these values in Eq (38), we get

$$\begin{array}{ccc} Var[hS\cos\left(\theta_{f}\right)] & = & b^{2}S^{2}\left[1-\frac{\pi }{2}{\left(\frac{\sin(\phi )}{\phi }\right)}^{2}+\frac{\sin(\phi )}{2\phi }\right] \end{array}$$ (39)
